# Social cognition and prefrontal hemodynamic responses during a working memory task in schizophrenia

**DOI:** 10.1038/srep22500

**Published:** 2016-03-01

**Authors:** Shenghong Pu, Kazuyuki Nakagome, Takeshi Yamada, Masashi Itakura, Takehiko Yamanashi, Sayaka Yamada, Mieko Masai, Akihiko Miura, Takahira Yamauchi, Takahiro Satake, Masaaki Iwata, Izumi Nagata, David L. Roberts, Koichi Kaneko

**Affiliations:** 1 Division of Neuropsychiatry, Department of Brain and Neuroscience, Tottori University Faculty of Medicine: 36-1 Nishi-cho, Yonago, Tottori, Japan; 2 National Center of Neurology and Psychiatry: 4-1-1 Ogawa-Higashi, Kodaira, Tokyo, Japan; 3 Division of Psychiatry, Kurayoshi Hospital, 43 Yamane, Kurayoshi, Japan; 4 Department of Psychiatry, The University of Texas Health Center at San Antonio, San Antonio, USA

## Abstract

Social cognition is an important determinant of functional impairment in schizophrenia, but its relationship with the prefrontal functional abnormalities associated with the condition is still unclear. The present study aimed to explore the relationship between social cognition and prefrontal function in patients with schizophrenia using 52-channel near-infrared spectroscopy (NIRS). Twenty-six patients with schizophrenia and 26 age-, gender-, and intelligence quotient-matched healthy controls (HCs) participated in the study. Hemodynamic responses in the prefrontal and superior temporal cortical regions were assessed during a working memory task using NIRS. Social cognition was assessed using the Social Cognition Screening Questionnaire (SCSQ). The observed hemodynamic responses were significantly reduced in the lateral prefrontal cortex (PFC), the frontopolar cortex, and temporal regions in subjects with schizophrenia compared to HCs. Additionally, lateral PFC hemodynamic responses assessed during the working memory task demonstrated a strong positive correlation with the SCSQ theory of mind (ToM) subscale score even after controlling for working memory performance. These results suggest that ToM integrity is closely related to lateral PFC functional abnormalities found in patients with schizophrenia. In addition, this study provides evidence to suggest that NIRS could be used to identify biomarkers of social cognition function in subjects with schizophrenia.

Recent meta-analyses have indicated that social cognitive function is markedly impaired in patients with schizophrenia^1^. In schizophrenia, social cognition may serve as a mediator between cognition and functional outcome^2^, wherein social cognition has even been suggested as a determinant of functional and behavioral outcomes^2^,^3^. Social cognition refers to the mental processes underlying social interactions, including the prediction, perception of and reaction to others’ intentions and behaviors^3^. In daily life, a large degree of social cognitive processing requires the real-time tracking and integration of information regarding an individual’s psychological characteristics, their mental and emotional states, and the relation of these across individuals—functions that overlap with traditional working memory function^4^. Working memory incorporates multiple cognitive mechanisms that enable the active maintenance and manipulation of information acquired from both internal and external sources. The ability to sustain functional social interactions requires the maintenance and manipulation of significant amounts of social information. Indeed, the “social brain hypothesis” suggests that the human brain evolved a larger relative size to meet increased social cognitive demands^5^. Given the evidence underlying the importance of working memory in the production of appropriate social behaviours^6^, this suggests that the brain regions supporting working memory function are essential for successful social interactions^4^.

Despite its central role in higher order cognition, relatively limited research has evaluated the contribution of working memory to social cognition. A few studies have suggested that social cognition, particularly theory of mind (ToM), requires efficient working memory function^7^,^8^,^9^. An important component of social cognition, ToM, or mentalizing, is the ability to understand and attribute mental states, including intentions, knowledge, and desires. Supporting the role of working memory in social cognition, complex mentalizing depends on the integrity of working memory^10^. Meyer *et al*.^4^,^11^ recently demonstrated that the maintenance and manipulation of social cognitive information recruits two distinct cognitive networks: the medial frontoparietal system associated with mental state reasoning^12^ and the lateral frontoparietal system associated with traditional cognitive working memory tasks^13^.

Working memory impairment is considered central to schizophrenia, and the prefrontal cortex (PFC), a region critical for working memory performance, has been demonstrated as a critical predictor of functional impairment in schizophrenia^14^. The hemodynamic responses underlying working memory processes have been widely investigated using neuroimaging (fMRI and PET) techniques^13^,^15^. In healthy subjects, the n-back task, used to evaluate working memory function, activated a bilateral network consisting of the ventrolateral PFC (VLPFC), dorsolateral PFC (DLPFC), frontopolar cortex (FPC), lateral premotor cortex, dorsal cingulate, and medial premotor cortices, in addition to the medial and lateral posterior parietal cortices^15^. In patients with schizophrenia, working memory deficits have been associated with dysfunction in the DLPFC in addition to interconnected regions^5^. Previous n-back task-based studies have indicated that patients with schizophrenia have impaired DLPFC activation^16^,^17^. Comparatively, other studies have reported that patients with schizophrenia demonstrate greater PFC activation compared to healthy controls (HCs), suggesting a shift to an inverted U-curve activity pattern^18^, or a compensatory response arising from preserved regions^17^,^18^,^19^.

The primary objective of the present study was to explore the relationship between working memory-related PFC activity and social cognition in patients with schizophrenia using multi-channel near-infrared spectroscopy (NIRS) imaging. We hypothesized that PFC hemodynamic responses associated with working memory would be correlated with social cognition deficits in patients with schizophrenia.

## Results

### General Demographics

The participants’ demographic data are displayed in Table 1.

### Task performance

The response sensitivity A’ and reaction time (RT) scores in the 2-back task during NIRS measurement were significantly worse in the schizophrenia group than HCs. (Table 1).

### Cognitive Activation

**Figure 1:**
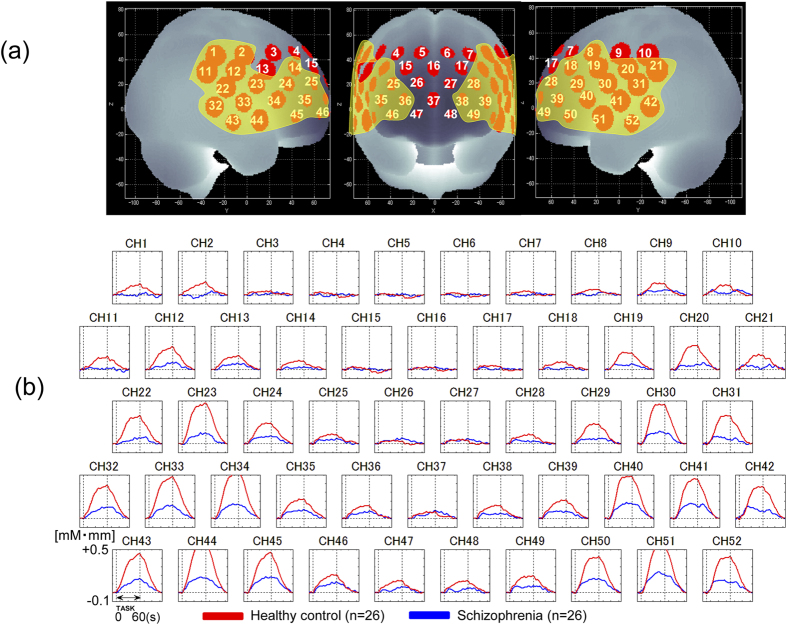
(**a**) Brain area in yellow corresponds to the near-infrared spectroscopy (NIRS) channels with significantly lower levels of activation in the schizophrenia than in the healthy control (HCs) (false discovery rate [FDR]-corrected *p* < 0.05, corrected with 52 channels). The locations of NIRS channels were probabilistically estimated and anatomically labeled in the standard brain space in accordance with Tsuzuki *et al*.^54^. (**b**) Grand averaged waveforms of oxygenated hemoglobin (oxy-Hb) during the working memory task (between two dotted vertical lines in each graph) in 52 channels over prefrontal and temporal regions measured by NIRS. Red and blue lines represent schizophrenia and HCs, respectively.

(Figure 1) To assess for the presence of significant activations in regions related to working memory, we compared the mean oxy-Hb changes between the pre-task (0-back) and 60-s task (2-back) periods. HCs demonstrated a significant increase in activity in the DLPFC (12 channels: ch4, ch7, ch8, ch14, ch17, ch18, ch24, ch25, ch28, ch29, ch35, and ch39), the VLPFC (8 channels: ch13, ch19, ch23, ch30, ch34, ch40, ch45, and ch50), the FPC (9 channels: ch26, ch27, ch36 to ch38, and ch46 to ch49) and temporal regions (10 channels: ch22, ch31 to ch33, ch41 to ch44, ch51, and ch52) (FDR-corrected *P* < 0.05, corrected for 52 channels). Comparatively, patients with schizophrenia demonstrated a significant increase in activity in the DLPFC (6 channels: ch24, ch25, ch28, ch29, ch35, and ch39), the VLPFC (6 channels: ch19, ch30, ch34, ch40, ch45, and ch50), regions of the FPC (6 channels: ch26, ch27, ch36, ch38, ch46, and ch49), and temporal regions (7 channels: ch33, ch41 to ch44, ch51, and ch52) (FDR-corrected *P* < 0.05, corrected for 52 channels).

Patients with schizophrenia exhibited significantly smaller increases in oxy-Hb than controls for 36 channels (ch1, ch2, ch8, ch11, ch12, ch14, ch18 to ch25, ch28 to ch36, ch38 to ch46, ch49 to ch52; FDR-corrected *P* < 0.05, corrected with 52 channels), which were predominantly distributed between the DLPFC, VLPFC, the FPC, and temporal regions (Fig. 1). The between-group differences for mean oxy-Hb changes remained significant after correcting for performance levels in 34 channels (ch1, ch2, ch11, ch12, ch14, ch18 to ch25, ch28, ch29, ch31 to ch36, ch38 to ch46, ch49 to ch52; FDR-corrected *P* < 0.05, corrected with 36 channels) with ANCOVA using RT and sensitivity A’ as a covariate to the mean oxy-Hb changes.

### Correlation Analyses

**Figure 2:**
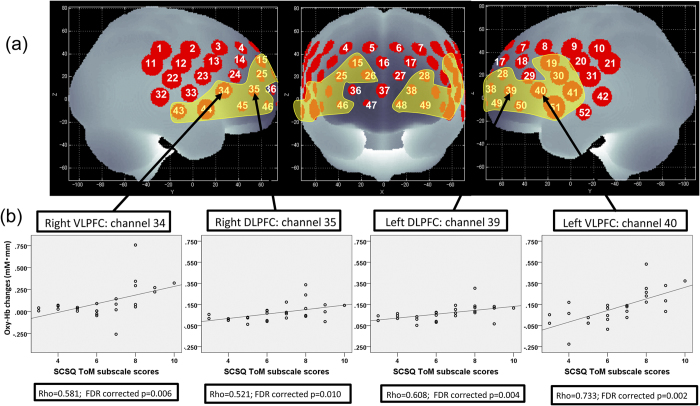
Cortical distribution displaying the areas of significant correlation between mean oxy-Hb changes and social cognition function (ToM subscale scores). (**a**) Brain area in yellow corresponds to the NIRS channels in which mean oxy-Hb changes demonstrate a significant correlation with social cognition (Social Cognition Screening Questionnaire [SCSQ] ToM subscale scores; Spearman’s correlation coefficient; FDR-corrected *p* < 0.05). (**b**) Scatter diagrams showing the relationship between SCSQ ToM subscale scores and mean oxy-Hb changes in channels 34 (right VLPFC), 35 (right DLPFC), 39 (left DLPFC), and 40 (left VLPFC). The locations of NIRS channels were estimated probabilistically and labeled anatomically in the standard brain space in accordance with Tsuzuki *et al*.^54^

In patients with schizophrenia, the mean oxy-Hb change demonstrated a significant positive correlation with Social Cognition Screening Questionnaire (SCSQ) ToM subscale scores in 20 channels (ch15, ch19, ch25, ch26, ch28, ch30, ch34, ch35, ch38 to ch41, ch43 to ch46, and ch48 to 51; rho = 0.475 to 0.782; FDR-corrected *P* < 0.05, corrected for 52 channels, Fig. 2) in the DLPFC, VLPFC, FPC, and temporal regions. No significant relationships were found with other subscales of the SCSQ and total scores (FDR-corrected *P* > 0.05, corrected for 52 channels).

Of the 20 channels correlated significantly with SCSQ ToM subscale scores in the schizophrenia group, multiple regression analyses revealed significant contributions for the SCSQ ToM subscale scores in 15 channels (β = 0.445 to 0.710; *P* < 0.05) (Table 2). Age, gender, and GAF scores did not significantly influence either working memory-related cortical activity or the correlation between ToM scores and the cortical activity. Significant contributions were identified for RT (task performance on the 2-back) in 5 channels (ch19, ch34, and ch43 to ch45), premorbid IQ in 1 channel (ch30), positive syndrome (PANSS) in 1 channel (ch41), general psychopathology (PANSS) in 2 channels (ch44 and ch51), daily dosage of antipsychotic drugs (CPZ: Chlorpromazine equivalent) in 5 channels (ch19, ch35, ch39, ch45, and ch50), verbal working memory (SCSQ) in 1 channel (ch19), metacognition (SCSQ) in 2 channels (ch39 and ch41), and hostility bias (SCSQ) in 1 channel (ch48), as shown in Table 2.

## Discussion

To our knowledge, this is the first study using multi-channel NIRS to identify a functional relationship between working memory-related hemodynamic responses in the frontotemporal regions and social cognition in patients with schizophrenia. Hb concentration changes in the prefrontal and temporal cortical surface areas were evaluated during a working memory task. Regional hemodynamic responses were significantly reduced in subjects with schizophrenia in the lateral PFC, FPC, and some temporal regions. Notably, lateral PFC hemodynamic responses during the working memory task correlated with SCSQ ToM subscale scores, even after controlling for working memory task performance and demographic factors. These results suggest that ToM function could be supported by lateral PFC activity in patients with schizophrenia, and that NIRS could potentially be used to identify biomarkers of social cognition function in subjects with schizophrenia.

Supporting this, studies have reported that the PFC functions not only in working memory, but also in the regulation of social and emotional behaviors^20^. More specifically, recent neuroimaging studies suggest that both the lateral and medial PFC are particularly important for ToM^21^,^22^,^23^,^24^. ToM is the mentalizing capacity to infer the mental states of others, including their thoughts, desires, and intentions^25^. Arguably, complex mentalizing depends on some form of working memory, since considering and attributing mental states requires one to maintain and manipulate information about the person (self or other) in order to draw a conclusion about their mental state. Furthermore, there is evidence that working-memory deficits might contribute to ToM impairment in remitted patients with schizophrenia^26^. Thus, because ToM requires the above working memory processes, the neural basis of ToM may include brain regions associated with them. Indeed, additional evidence suggests that the VLPFC is involved in affective ToM processing (e.g., the faux pas task)^21^,^27^,^28^,^29^, while the DLPFC is involved in the processing of cognitive ToM (e.g., the false belief task)^30^,^31^. These studies provide converging evidence that a functioning ToM is supported by regions outside of those traditionally considered to comprise the ToM network, which consists of such as posterior superior temporal sulcus, inferior frontal and parietal cortex, and anterior rostral medial prefrontal cortex^32^, and that the lateral PFC is one of those newly identified regions^24^,^33^. Furthermore, lesion studies have highlighted the key role of prefrontal and frontal brain areas in ToM function^34^,^35^,^36^,^37^,^38^. Indeed, a recent report suggested that ToM performance was significantly compromised in patients with lateral PFC damage compared to their control group counterparts^39^.

The lateral PFC also is involved in the representation and integration of goals and reward information^40^. It maintains goal-relevant information during working memory^15^, updates this representation as during task switching^41^, and arbitrates between conflicting goals during decision making^41^,^42^. In light of the present findings, the hemodynamic response observed in the lateral PFC region during working memory tasks may be associated with social cognition, especially ToM, in patients with schizophrenia.

The present results suggest that lateral PFC function is relevant to ToM performance above and beyond its relevance to traditional working memory. RT in the working memory task demonstrated a significant correlation with hemodynamic responses in 5 channels including DLPFC, VLPFC and temporal regions, Also, SCSQ performance is susceptible to influence by verbal memory deficits (see supplementary Table S1)^43^, Thus, we included working memory task performance (sensitivity A’ and RT) and the SCSQ verbal working memory subscale scores and as covariates in the multiple regression analysis to identify any significant interactions between working memory ability and SCSQ ToM subscale data with PFC functional abnormalities. Lateral PFC hemodynamic responses during the working memory task correlated with SCSQ ToM subscale scores even after controlling for these two working memory measures and demographic factors. Therefore, it seems reasonable to consider that lateral PFC function is relevant to ToM performance beyond its relevance to traditional working memory.

Our findings need to be interpreted within the context of the study limitations. First, multichannel NIRS has limited spatial resolution compared to fMRI and PET. However, a recent MRI–NIRS combination study, which used a probabilistic method to register the NIRS data to the Montreal Neurological Institute (MNI) coordinate space, suggested that the errors of spatial estimation were approximately 10 mm^44^,^45^. These results suggest that multichannel NIRS can to some degree detect sub-region-specific activation in the PFC. However, even though NIRS is a method well-suited to obtain physiological data from the cerebral cortex, it was not possible to completely determine the definite locations of cortical regions. Although the ToM subscale score in the SCSQ was assumed to rely more on the cognitive processing of ToM, it was considered implausible to disentangle the locations between the dorsolateral and ventrolateral PFC. Therefore, different PFC areas might have partly contributed to the observed association. In future studies it would be appropriate to add other ToM tasks that are more relevant to affective processing, such as the faux pas task, to distinguish the role of PFC in cognitive versus affective components of ToM.

Another limitation related to the inherent characteristics of NIRS is the inability to assess other brain regions essential to social cognitive processing that are distant from the scalp, such as the medial PFC. Secondly, although we did not establish a relationship between oxy-Hb signals and the duration of illness or dosages of medication in the patients with schizophrenia, most of the patients in this study were chronically ill and medicated. Therefore, to rule out the effects of medication, future studies are warranted using first-episode and/or drug-naïve patients with schizophrenia to control for this. Thirdly, we did not find a significant correlation between the sensitivity A’ of the 2-back task and prefrontal activity. With the aim of simplifying the task so as to reduce the influence of set-shifting processing only one block of the 2-back task was used, which in turn may have reduced the validity of the sensitivity A’ variable owing to the small number of targets. Moreover, we did not administer the SCSQ in normal controls, although in a previous study, we found a reliable discriminatory effect between patients and normal controls using SCSQ total scores and ToM subscale scores^46^. Finally, the sample was obtained from the outpatient population of Tottori University hospital, and thus might not be representative of the general population of patients with schizophrenia.

In conclusion, despite these limitations, our study indicated that ToM function might be related to lateral PFC function abnormalities in patients with schizophrenia, and that NIRS has the potential to identify biomarkers of social cognition function in subjects with schizophrenia. In addition, since the SCSQ ToM subscale places a relatively high demand on working memory, it might well represent social cognition in day-to-day social life where a high demand on working memory is involved. Further studies with a larger sample size will be required to verify these findings.

## Material and Methods

### Participants

Twenty-six patients (male, 8; female, 18) diagnosed with schizophrenia based on the Diagnostic and Statistical Manual of Mental Disorders, fourth edition (DSM-IV), using the Mini-International Neuropsychiatric Interview (MINI)^46^, were recruited for the present study. All participants had been prescribed antipsychotic medication (atypical). Daily doses of all antipsychotics were converted to the equivalent dose of chlorpromazine^47^. On the day of NIRS evaluation, psychiatric symptoms and global functioning were evaluated by psychiatrists (I.N., and K.K.) using the Positive and Negative Syndrome Scale (PANSS) and the Global Assessment of Functioning (GAF) Scale, respectively.

Patients with comorbid neurological illness, previous traumatic brain injury with any known cognitive consequences or loss of consciousness for more than 5 min, a history of electroconvulsive therapy, or alcohol/substance abuse or addiction (except nicotine) were excluded.

Healthy individuals were matched to patients with respect to age, gender, and premorbid intelligence quotient (IQ) as controls for the present study. Premorbid IQ was estimated using the Japanese version of the National Adult Reading Test^48^. The inclusion criteria for controls were similar to those for the patient sample, although controls were also required to have no previous or current diagnosis of psychiatric illness. Twenty-six individuals (male, 8; female, 18) meeting these criteria were selected to participate in the study.

All participants were right-handed according to the Edinburgh Handedness Inventory^49^ and were native Japanese speakers. All participants gave informed written consent. The study was approved by the Ethics Committee of Tottori University Faculty of Medicine, and the investigation was carried out in accordance with the latest version of the Declaration of Helsinki.

### Social cognitive measures

The Social Cognition Screening Questionnaire (SCSQ)^43^,^50^ contains five subscales: verbal working memory, schematic inference, ToM, metacognition, and hostility bias. The task comprised 10 short vignettes presenting an interaction between a fictional character and the study participant. Each vignette was read aloud by the tester, and repeated once at the subject’s request. The subject then answered three Yes-or-No questions about the vignette, which were used to derive subscale scores for the SCSQ. ToM tasks were essentially verbal in nature and designed to assess both ToM and hostile attributional bias. The three questions were presented in random order, with the last followed by a self-rating of the participant’s confidence in the accuracy of their final Yes-or-No answer (‘very sure’, ‘pretty sure’, ‘a little unsure’, or ‘not sure at all’). The sum of correct answers for the verbal working memory, schematic inference, and ToM subscales was calculated (range 0–10; higher scores indicated better performance). Scoring for the hostility bias scale incorporated the sum of instances in which the subject erroneously inferred that the vignette character had negative thoughts or feelings toward the subject (range 0–5; higher scores indicate greater bias). With regard to metacognition scores, if the subject answered correctly on the last Yes-or-No question, a score of 1 was given. If the subject answered incorrectly on the last question a score of 0 was given if he/she answered that he/she was ‘very sure’, 0.33 for ‘pretty sure’, 0.66 for ‘a little unsure’, and 1 for ‘not sure at all’. The total metacognition score was obtained by summing the scores for the 10 vignettes (range 0–10; higher scores indicate better metacognitive ability). The SCSQ total score was calculated as the sum of all the subscales except for the hostility bias scale, because the items used for calculating this scale overlapped with those used for the ToM scale. The SCSQ was administered to only the schizophrenia patients (see supplementary text for a sample item).

### Working Memory Task

We used a 2-back task with a blocked periodic baseline–activation–baseline design to activate brain regions implicated in the maintenance and manipulation components of verbal working memory, as originally described by Cohen *et al*.^51^ Two contrasting conditions were visually presented in 60 s periods to subjects on a computer screen placed approximately 1 m away from the subjects’ eyes. The working memory task consisted of a 60 s pre-task period, a 60 s 2-back task period, and a 60 s post-task period (one 180 s block, see supplementary Figure S1 for details). During the pre- and post-task periods, subjects viewed a series of numbers (0–9) presented one at a time, and were required to press a button with their right index finger whenever the number “9” appeared (0-back). During the 60-s task period, subjects again viewed a series of numbers (0–9), and were required to press a button with their right index finger if the currently presented number was the same as that presented two trials previously (2-back, e.g., 5-1-5 but not 2-6-3-2 or 2-7-7). Each period comprised 25 stimuli (five targets, stimulus duration 1.8 s, stimulus onset asynchrony = 2.3 s). Behavioral performance for the 2-back task was monitored and assessed in terms of reaction time to target figures and sensitivity A’^52^. Sensitivity A’ is an index of information processing ability using both “hit rate (HR)” and “false alarm rate (FAR)” for calculation expressed as the equation below:


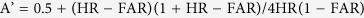


A high A’ value indicates greater information processing ability. All subjects received a brief period of identical training to ensure that they understood the rules of the task prior to assessment.

### NIRS methodology

A 52-channel NIRS (ETG-4000, Hitachi Medical Co.) machine was used to measure relative changes in oxygenated hemoglobin (oxy-Hb) and deoxygenated hemoglobin (deoxy-Hb) at two wavelengths (695 and 830 nm) of infrared light, based on the modified Beer–Lambert law^44^. The NIRS probe comprised 3 × 11 arrays with 17 emitters and 16 detectors. The probes of the NIRS machine were placed on the frontotemporal region of each participant, with the mid-column of the probe located over Fpz and the lowest probes located along the T3-Fp1-Fpz-Fp2-T4 line, in accordance with the International 10–20 Placement System used for electroencephalography. The distance between pairs of source-detector probes was set at 3 cm, and each measurement area between pairs of source-detector probes was defined as a “channel” (ch). The machine records regions at a depth of 2–3 cm below the scalp, which corresponds with the surface of the cerebral cortex. The probe arrangement used enabled the measurement of Hb values from the surface regions of both the PFC and temporal regions. The correspondence between the NIRS channels and cortical anatomy has been confirmed in a multi-subject study^53^. Spatial information from each channel was estimated using functions from the Functional Brain Science Laboratory at Jichi Medical University in Japan (http://www.jichi.ac.jp/brainlab/virtual_reg.html)^54^.

The sampling frequency was 10 Hz. To examine 2-back task-related activation, data were analyzed using the “integral mode” installed on the NIRS machine. Herein, the pre-task baseline was calculated as the mean over the 10 s period prior to the task period, and the post-task baseline was calculated as the mean over the 5 s period after the 50 s post-task period (see supplementary Figure S1 for details). Linear fitting was applied to the data recorded between these two baselines. A moving-average method, using a 5 s window, was applied to remove any short-term motion artifacts. In addition, noise related to body-movement artifacts (no signal, high frequency, and low frequency) were rejected using the algorithm published in Takizawa *et al*.^55^

Mean changes in oxy-Hb (as opposed to deoxy-Hb) measured during the 2-back task were used as an index of cortical activity, because oxy-Hb better reflects this activity and correlates suitably with fMRI blood oxygenation level-dependent (BOLD) signals^56^.

### Statistical Analyses

Statistical analyses were performed using SPSS Statistics 19.0 (Tokyo, Japan).

A false discovery rate (FDR)-based procedure was adopted for correcting for multiple tests in the between-groups comparisons. For the correlational analyses of the 52 channels, we identified channels for which the rho values reached an FDR-corrected significance level of *P* < 0.05. We set the value of q, specifying the maximum FDR at 0.05, so that no more than 5% of channels registered as falsely positive on average^57^.

Categorical variables were compared using the chi-square test. In all groups, the clinical variables that fit the normal distribution were compared using t-tests, while the Mann-Whitney U-test was used for clinical variables that were not normally distributed. Mean oxy-Hb changes during the task period were compared between groups using t-tests. When there was a significant between-group difference in the performance level (RT and sensitivity A’), we performed additional analyses of co-variance (ANCOVA) using the performance level (RT and sensitivity A’) as covariates to the oxy-Hb changes, also applying FDR correction. To examine the relationships between mean oxy-Hb changes and social cognitive function (total and subscale scores) in the schizophrenia group, we calculated Spearman’s rho correlation coefficients for this data. In addition, to elucidate the independent contributions of mean oxy-Hb changes to social cognition in those channels that reported significant correlations, we performed stepwise multiple regression analyses for the schizophrenia group. In these analyses, mean oxy-Hb changes was set as the dependent variable, and other potential confounding variables such as age, gender (dummy parameterized, male = 1, female = 0), premorbid IQ, task performance on the 2-back (RT and Sensitivity A’), GAF score, PANSS score, and daily dosage of antipsychotic drugs in the analyses of the schizophrenia group, were controlled for with a probability of F for conservative entry and removal criteria of P = 0.05 and 0.2, respectively. For significant findings, effect sizes were indicated using the standardized regression coefficient (β).

## Additional Information

**How to cite this article**: Pu, S. *et al*. Social cognition and prefrontal hemodynamic responses during a working memory task in schizophrenia. *Sci. Rep.* **6**, 22500; doi: 10.1038/srep22500 (2016).

## Supplementary Material

Supplementary Information

## Figures and Tables

**Table 1:** Demographics and clinical characteristics of participants.

	Patients with schizophrenia n = 26 (mean ± SD)	Healthy controls n = 26 (mean ± SD)	Between-group comparison
Age, years	31.6 ± 8.7	31.2 ± 6.9	t (df = 50) = 0.177, *P* = 0.861
Gender, women/men	18/8	18/8	X2 = 0, *P* = 1.000
Edinburg handedness inventory (%)	97.7 ± 11.8	94.7 ± 8.7	t (df = 50) = 1.048, *P* = 0.300
Estimated premorbid IQ	99.2 ± 11.1	100.5 ± 9.2	t (df = 50) = −0.475, *P* = 0.637
Task performance (2-back): Reaction time (ms)	867.8 ± 254.7	630.8 ± 181.3	U = 137, *P* < 0.001
Sensitivity A’	0.882 ± 0.149	0.988 ± 0.021	U = 195, *P* < 0.005
Age at onset, years	21.5 ± 8.3	–	–
Duration of illness, years	10.0 ± 6.1	–	–
PANSS Total	63.4 ± 16.0	–	–
Positive	13.9 ± 4.1	–	–
Negative	17.9 ± 5.3	–	–
General psychopathology	31.6 ± 8.4	–	–
GAF	52.6 ± 9.4	–	–
SCSQ Verbal memory	8.1 ± 1.1	–	–
Schematic inference	7.8 ± 1.6	–	–
Theory of mind	6.5 ± 1.9	–	–
Metacognition	7.9 ± 2.1	–	–
Hostility bias	1.4 ± 1.1	–	–
Total	30.3 ± 3.1	–	–
Chlorpromazine equivalent dose, mg/day	30.3 ± 3.1	–	–

Abbreviations: IQ, Intelligence Quotient; PANSS, Positive and Negative Symptom Scale; GAF, Global Assessment of Functioning; SCSQ, Social Cognition Screening Questionnaire.

**Table 2:** Summary of stepwise multiple regression analysis in channels demonstrating a significant correlation with SCSQ ToM subscales scores in schizophrenia patients.

No. of channels^a^,^b^	R^2^	Adjusted R^2^	Independent Variables		Other Factors
						ToM	
						β	*P*
Ch15^a^	0.241	0.208	0.491	0.013	
Ch19^a^	0.449	0.374	0.629	0.005	SCSQ (verbal working memory): *β* = −0.388, *P* = 0.031; RT: *β* = −0.331, *P* = 0.031
Ch25^a^	0.406	0.354	0.617	0.005	CPZ: *β* = −0.388, *P* = 0.029
Ch26^b^					
Ch28^a^	0.240	0.208	0.490	0.004	
Ch30^a^	0.269	0.234			IQ: *β* = 0.519, *P* = 0.011
Ch34^a^	0.563	0.524	0.572	0.001	RT: *β* = −0.565, *P* = 0.001
Ch35^a^	0.548	0.508	0.637	< 0.001	CPZ: *β* = −0.579, *P* = 0.001
Ch38^a^	0.211	0.178	0.459	0.018	
Ch39^a^	0.433	0.384	0.612	0.001	CPZ: *β* = −0.451, *P* = 0.011; SCSQ (metacognition): *β* = −0.354, *P* = 0.027
Ch40^a^	0.561	0.522	0.539	0.001	
Ch41^a^	0.398	0.338			SCSQ (metacognition): *β* = −0.473, *P* = 0.013; PANSS (positive): *β* = −0.379, *P* = 0.042
Ch43^a^	0.517	0.471	0.499	0.004	RT: *β* = −0.579, *P* = 0.001
Ch44^a^	0.699	0.644	0.614	< 0.001	RT: *β* = −0.539, *P* < 0.001; PANSS (general psychopathology): *β* = −0.321, *P* = 0.016
Ch45^a^	0.652	0.603	0.707	< 0.001	RT: *β* = −0.400, *P* = 0.006; CPZ: *β* = −0.385, *P* = 0.009
Ch46^b^					
Ch48^a^	0.163	0.128			SCSQ (hostility bias): *β* = −0.404, *P* = 0.041
Ch49^a^	0.198	0.164	0.445	0.023	
Ch50^a^	0.517	0.475	0.710	< 0.001	CPZ: *β* = −0.402, *P* = 0.014
Ch51^a^	0.497	0.453	0.586	0.001	PANSS (general psychopathology): *β* = −0.396, *P* = 0.013

Note: SCSQ, Social Cognition Screening Questionnaire; ToM, theory of mind; No., number; Ch, channels; RT, reaction time; CPZ, chlorpromazine; IQ, intelligence quotient; PANSS, Positive and Negative Syndrome Scale.

^a^SCSQ (five subscales: verbal working memory, schematic inference, ToM, metacognition, and hostility bias), age, gender, premorbid IQ, task performance on the 2-back (RT and Sensitivity A’), Global Assessment of Functioning (GAF), PANSS score, and daily dosage of antipsychotic drugs were included in the multiple linear regression analysis.

^b^SCSQ (five subscales: verbal working memory, schematic inference, ToM, metacognition, and hostility bias), age, gender, premorbid IQ, task performance on the 2-back (RT and Sensitivity A’), GAF, PANSS score, and daily dosage of antipsychotic drugs did not show significant contributions to cortical activity in Ch26 and Ch46.
