# Supplementary material for: Social cognition and prefrontal hemodynamic responses during a working memory task in schizophrenia
Source: Sci Rep. 2016 Mar 1;6:22500. doi: 10.1038/srep22500 (PMC4772542; doi:10.1038/srep22500)
Supplement: Supplementary Information [file srep22500-s1.doc]

**Social cognition and prefrontal hemodynamic responses during a working memory task in schizophrenia**

**Shenghong Pu^a,*^, Kazuyuki Nakagome^b^, Takeshi Yamada^c^, Masashi Itakura^a^, Takehiko Yamanashi^a^, Sayaka Yamada^c^, Mieko Masai^a^, Akihiko Miura^a^, Takahira Yamauchi^a^, Takahiro Satake^a^, Masaaki Iwata^a^, Izumi Nagata^a^, David L Roberts^d^, Koichi Kaneko^a^**

^a^Division of Neuropsychiatry, Department of Brain and Neuroscience, Tottori University Faculty of Medicine: 36-1 Nishi-cho, Yonago, Tottori, Japan

^b^National Center of Neurology and Psychiatry: 4-1-1 Ogawa-Higashi, Kodaira, Tokyo, Japan

^c^Division of Psychiatry, Kurayoshi Hospital, 43 Yamane, Kurayoshi, Japan.

^d^Department of Psychiatry, The University of Texas Health Center at San Antonio, San Antonio, USA.

^*^Corresponding author: Shenghong Pu, [pshh0517@yahoo.co.jp](mailto:pshh0517@yahoo.co.jp)

Division of Neuropsychiatry, Department of Brain and Neuroscience, Tottori University Faculty of Medicine, 36-1 Nishi-cho, Yonago, Tottori 683-8504, Japan. Tel.: +81 859 38 6547; Fax: +81 859 38 6549.

**Supplementary Information:**

**Supplementary Table S1.**

**Spearman's rho between SCSQ scores and working memory ability**

**Supplementary text.**

**Sample item of Social Cognition screening Questionnaire (SCSQ)**

*Imagine that one night you accidentally cook too much spaghetti and tomato sauce for dinner. You decide to call your friend and invite him to come over and eat with you. He doesn’t answer the phone, so you leave a message. You wait around until the food is cold, but your friend never shows up for dinner. Finally, you eat cold noodles and then go out for a walk. While walking, you see your friend come out of a movie theater with another person, smiling and laughing. When they see you, your friend looks surprised.*

A. Did you make pizza for dinner? (Y/N) [verbal memory]

B. Did you eat dinner early that night? (Y/N) [schematic inference]

C. Was your friend hoping that you would not see them at the movie theater? (Y/N) [theory of mind / hostility bias]

D. How sure are you that your last answer (C) was right?

(0=not at all sure; 1=a little unsure; 2=pretty sure; 3=very sure) [metacognitive overconfidence]


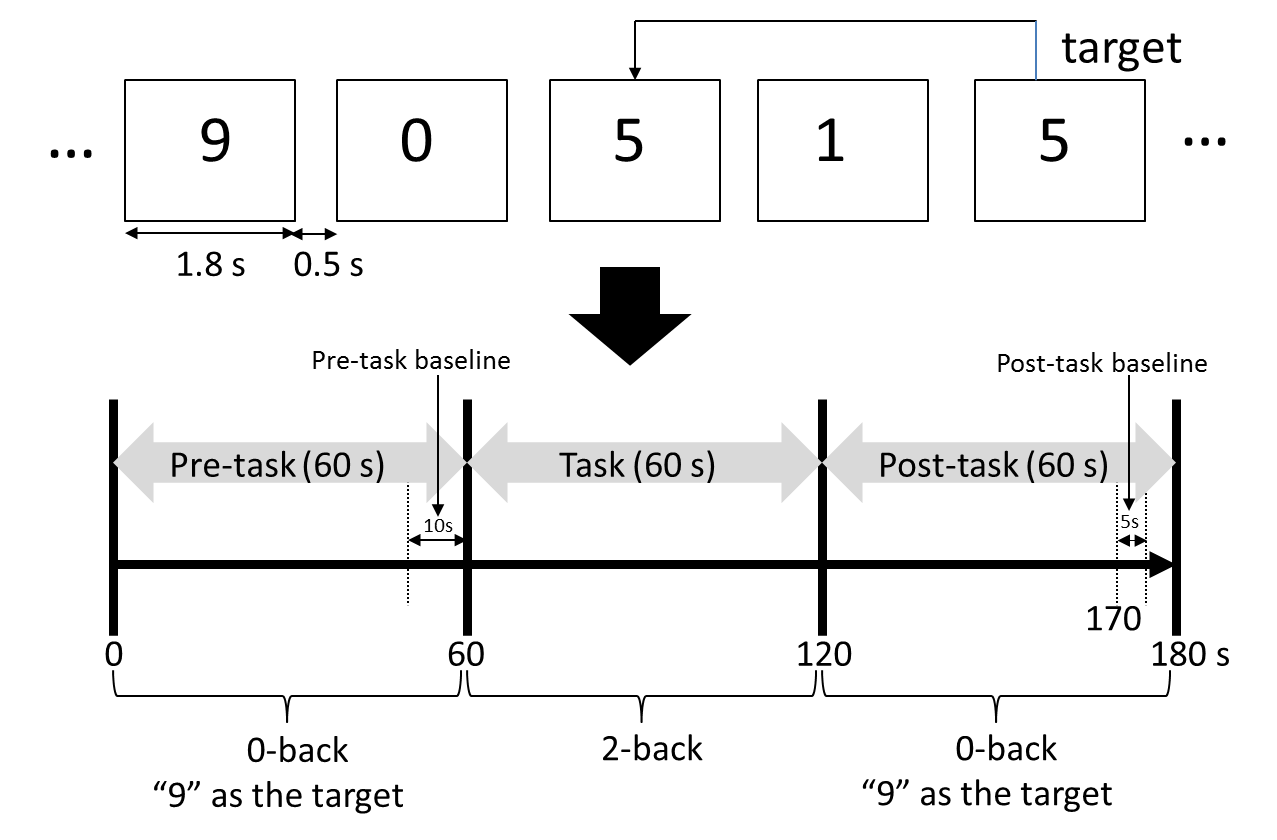


**Supplementary Figure S1.** The working memory task paradigm and the task segments used for statistical analysis in near-infrared spectroscopy (NIRS) measurements.
